# Prehabilitation exercise therapy for cancer: A systematic review and meta‐analysis

**DOI:** 10.1002/cam4.4021

**Published:** 2021-06-10

**Authors:** Christina M. Michael, Eric J. Lehrer, Kathryn H. Schmitz, Nicholas G. Zaorsky

**Affiliations:** ^1^ Penn State College of Medicine Hershey PA USA; ^2^ Department of Radiation Oncology Icahn School of Medicine at Mount Sinai New York NY USA; ^3^ Department of Radiation Oncology Penn State Cancer Institute Hershey PA USA; ^4^ Department of Public Health Sciences Penn State College of Medicine Hershey PA USA

**Keywords:** colorectal cancer, lung cancer, meta‐analysis, surgery, surgical therapy

## Abstract

**Objective:**

The purpose of this study was to determine the impact of prehabilitation exercise intervention with respect to (1) acceptability, feasibility, and safety; and (2) physical function, measured by 6‐minute‐walk test (6MWT).

**Data sources:**

PRISMA guidelines were used to systematically search PubMed, Embase, and CINAHL databases evaluating prehabilitation exercise interventions.

**Study selection:**

The inclusion criteria were studies investigating patients who underwent surgery for their cancer and underwent prehabilitation exercise.

**Data extraction and synthesis:**

Guidelines were applied by independent extraction by multiple observers. Data were pooled using a random‐effects model.

**Main outcome(s) and measure(s):**

Acceptability, feasibility, and safety rates were calculated. 6MWT (maximum distance a person can walk at their own pace on a hard, flat surface, measured in meters, with longer distance indicative of better performance status) was compared using two arms using the DerSimonian and Laird method.

**Results:**

Objective 1. Across 21 studies included in this review, 1564 patients were enrolled, 1371 (87.7%) accepted the trial; of 1371, 1230 (89.7% feasibility) completed the intervention. There was no grade 3+ toxicities. Objective 2. Meta‐analysis of five studies demonstrated a statistically significant decrease in 6MWT distance postoperatively in the control group (mean difference = +27.9 m; 95% confidence interval (CI): 9.3; 46.6) and a significant improvement postoperatively in the prehabilitation group (mean difference = −24.1 m; 95% CI: −45.7; −2.6). Meta‐analysis demonstrated improvements in 6MWT distance 4–8 weeks postoperatively in the prehabilitation group compared to the control group (mean difference = −58.0 m, 95% CI: −92.8; −23.3).

**Conclusions and relevance:**

Prehabilitation exercise for cancer patients undergoing surgery was found to be safe, acceptable, and feasible with a statistically significant improvement in the 6MWT, indicating that prehabilitation can improve postoperative functional capacity.

## Graphical abstract

Prehabilitation exercise for cancer patients undergoing surgery was found to be safe, acceptable, and feasible.In this meta‐analysis, panel A shows that there is a 27.9 m c post operatively in the control group. Panel B shows that there is a 24.1 m increase in distance walked during the 6MWTpostoperatively in the prehab group. Panel C shows that the two groups (control andprehab) have the same 6MWT distance before randomization. Panel D shows that prehab intervention improved the 6MWT by 58.0 m, suggesting that prehabilitation can improve postoperative functional capacity.

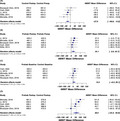

## 1 INTRODUCTION

Physical activity is becoming increasingly recognized as a valuable tool for improvement of functional capacity in cancer patients during treatment and rehabilitation.^1^, ^2^, ^3^, ^4^ However, little is known about the effect of preoperative exercise‐based conditioning, commonly termed “prehabilitation” or “prehab”.^5^ Several studies demonstrated varying results in terms of acceptability, feasibility, and postoperative patient function. This variation could be due to factors such as heterogenous patient populations at baseline or variations in cancer type in a patient population or outcomes. Currently, cancer guidelines from the National Comprehensive Cancer Network (NCCN) do not discuss prehab as a possible option for cancer patients.

The 6‐minute walk test (6MWT) has been found to be a prognostic factor for survival,^6^ and it is a common way to evaluate the impact of prehab on postoperative survival. For reference, the 6MWT is measures the distance (in meters) an individual is able to walk over a total of six minutes on a hard, flat surface. The individual is allowed to walk at their own pace rest as needed as they traverse back and forth along a marked walkway. A farther distance walked is more favorable than a shorter one. Studies that have assessed 6MWT demonstrated its value in predicting prognosis in functional capacity and overall patient survival of patients. In healthy adult subjects (40–80 years of age) the mean 6MWT distance was 571 ± 90 m with older subjects walking shorter distances than younger.^7^ Additionally, the 6MWT has been established as an independent and convenient prognostic factor of surgically treated non‐small‐cell lung cancer.^3^, ^6^, ^8^, ^9^

Given this known prognostic data and in view of the lack of data on the value of prehabilitation in cancer patients undergoing surgery, we aimed to create a systematic review and meta‐analysis to determine the role of prehabilitation exercise as a means of enhancing the physical condition of cancer patients during recovery from surgery. The objectives were to assess the (1) acceptability, feasibility, and safety of prehab therapy; and (2) physical function, measured by 6MWT. In addition, we discuss other outcome measures of functional capacity, such as postoperative pulmonary complications and the cardio‐pulmonary exercise test. The results of this study may be used to support prehab in the guidelines of cancer therapy.

## 2 METHODS

### 2.1 Eligibility criteria

Inclusion criteria for literature selection was defined using the Population, Intervention, Control, Outcomes, Study Design method (Table 1). The inclusion criteria for the qualitative systematic review were: (1) patients who underwent surgery for their cancer and were randomized to prehabilitation. For the meta‐analysis: (1) 6MWT distance reported at baseline and at 4–8 weeks postoperatively. Exclusion criteria were: (1) works involving non‐human subjects; (2) retrospective studies, (3) protocols, (4) studies without exercise prehabilitation, (5) systematic reviews and meta‐analyses (though these were searched to find eligible articles); (6) abstract alone; (7) works not published in English; (8) unfinished manuscripts. No studies were blinded due to the nature of the study.

**TABLE 1:** Population, Intervention, Control, Outcome, Study Design (PICOS) inclusion criteria

Population	Patients with cancer
Intervention	Prehabilitation exercise: strength, cardiovascular/aerobic, yoga/stretching
Control	Any control group with no prehabilitation, including standard of care therapy; or no control group
Outcomes	
Objective 1: Acceptability, feasibility, safety	Acceptability was defined as: (the number of patients agreeing to perform prehab +control)/(the number of participants screened and approached).Feasibility was defined as: (number of patients who completed prehab +control)/(number agreeing to perform prehab +control). Safety was defined as freedom from any CTCAE grade three or higher event, attributable to the addition of prehabilitation
Objective 2: Patient reported outcomes and physical function	6MWT, measured in meters
Other (if reported)	Post‐op pulmonary complications, CPET/VO2, METS/CHAMPS, physical function/strength, mood/depression/anxiety, QOL, length of stay/readmission rates
Study design	All prospective and retrospective randomized control trials, >10 patients, with one or more arms

Abbreviations: 6MWT, 6‐minute walk test; CHAMPS, Community Health Activities Model Program for Seniors; CPET, cardiopulmonary exercise testing; CTCAE, Common Terminology Criteria for Adverse Events; METs, metabolic equivalents; QOL, quality of life; VO2, VO2 max testing.

### 2.2 Information sources

A comprehensive literature search was performed using MEDLINE via PubMed, Embase via Elsevier, and CINAHL via EBSCO. Databases were searched from December 2018 to March 2019, with one article found and included in January 2020.

### 2.3 Search terms/Keywords

The following combination of keywords were searched: preoperative, presurgical, prehabilitation, prior to surgery, exercise, cycling, aerobic, cardiovascular, motion, movement, flexibility, walking, training, and sports. The full electronic search strategy for PubMed can be found in the supplementary material.

### 2.4 Process for study selection

Screening yielded 30 studies. 9 out of these 30 were excluded, leaving 21 for inclusion in qualitative analysis. Of these 21, 13 studies measured 6MWT‐ of these, five papers included the data necessary to be utilized for quantitative meta‐analysis. Articles were obtained by a medical student and reviewed with two MD, MS clinicians in the fields of oncology and epidemiology. One author (the medical student) was responsible for screening abstracts using PICO criteria. If a study's abstract fit PICO criteria, the whole article was then reviewed. Studies that were questionable were discussed with the senior authors as needed.

### 2.5 Process for data extraction

From the 21 studies characteristics of patients (e.g., age), cancer (e.g., disease site, and stage), treatment (e.g., surgery), prehabilitation exercise therapy (e.g., intensity, frequency, and supervision), patient reported outcomes, physical function, toxicity, and other outcomes were coded. Information on the studies and the locations they took place can be found in Table S4. The primary author abstracted the data into a database. Data were then reviewed as needed by senior authors. There was one consistent form of data abstraction.

### 2.6 Risk of bias assessment

Risk of bias was assessed using a Cochrane risk of bias assessment. Domains addressed include: random sequence generation (selection bias), allocation concealment (selection bias), blinding of participants and personnel (performance bias), blinding of outcome assessment (detection bias), incomplete outcome data (attrition bias), and selection reporting (reporting bias).

### 2.7 Data synthesis/statistical analysis methods

For objective 1, acceptability was defined as: (the number of patients agreeing to perform prehabilitation+control)/(the number of participants screened and approached). Feasibility was defined as: (number of patients who completed prehabilitation+control)/(number agreeing to perform prehabilitation+control). Dropout was defined as the number of patients who did not complete the study intervention, including those who died during the course of the study. Safety was defined as freedom from any Common Terminology Criteria for Adverse Events (CTCAE) grade three or higher event, attributable to the addition of prehabilitation exercise, per the assessment of the authors of the primary study.

A greater distance in 6MWT is superior and has demonstrated a moderate to strong association with higher maximum exercise capacity, perceived physical function, and has been proposed as a prognostic factor for survival in patients with lung cancer.^8^ Given this, for objective 2, a meta‐analysis was performed using the 6MWT to determine the impact of prehabilitation exercise intervention in cancer patients undergoing surgery. Details are shown in Figure S1. Briefly, two groups were randomized and baseline 6MWT distance (meters) was established for the prehab group and the control group. The prehabilitation then received prehabilitation exercise therapy prior to surgery and both groups underwent standard rehabilitation postoperatively. The two groups subsequently underwent the 6MWT 4–8 weeks postoperatively and their distances were compared to each other.

Of the 21 studies from the systematic review, 13 measured functional capacity using the 6MWT.^5^, ^10^, ^11^, ^12^, ^13^, ^14^, ^15^, ^16^, ^17^, ^18^, ^19^ Five of these 13 included necessary 6MWT data (mean distances, standard deviations, and number of patients) that allowed for a meta‐analysis to be performed.^10^, ^12^, ^13^, ^15^, ^19^ The authors of Gillis, 2014 were contacted to obtain standard deviation data for meta‐analysis. Table S2 demonstrates other outcomes measured to assess patients’ functional capacities during treatment and rehabilitation including: postoperative pulmonary complications, cardiopulmonary exercise testing (CPET), VO_2_ max testing, metabolic equivalents (METs), Community Health Activities Model Program for Seniors (CHAMPS), physical function/strength, mood, and quality of life (QOL).^5^, ^10^, ^11^, ^12^, ^13^, ^14^, ^15^, ^16^, ^17^, ^18^, ^19^, ^20^, ^21^, ^22^, ^23^, ^24^, ^25^, ^26^, ^27^

Statistical analyses were conducted using R Studio Version 1.1.383 (Boston, MA).^28^ The Meta‐Analysis for R (metafor) package version 2.0‐0 was used to conduct the meta‐analyses and tests for heterogeneity. The DerSimonian and Laird method was used to calculate between study variances using a random effects model.^29^ The mean difference of mean distance walked during postoperative 6MWT between the control and prehab groups was used as the effect size. A schematic of this can be seen in Figure S1. A random effects model was chosen over its fixed effects counterpart, as these studies were performed over the course of several years, among different populations. Furthermore, a random effects model is often the preferred technique when performing a meta‐analysis to guide patient treatment decisions.^30^, ^31^ Overall summary estimates for each of the outcome measures were depicted on forest diagrams with their associated 95% confidence interval.

Heterogeneity was assessed using both the I^2^ statistic^32^ and Cochran Q‐test.^33^ Significant heterogeneity was considered to be present if I^2^ >50% and the *p*‐value of the Q‐test was <0.10.

## 3 RESULTS

### 3.1 Study selection

**FIGURE 1:**
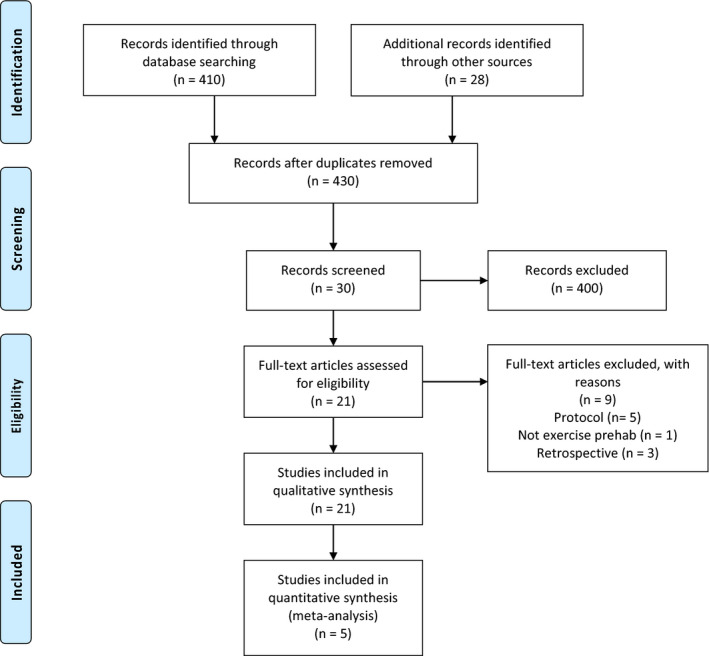
PRISMA

Additionally, the Preferred Reporting Items for Systematic Reviews and Meta‐analyses (PRISMA) selection algorithm (Figure 1) was designed. Guidelines from the PRISMA checklist (Figure 1) were followed.

### 3.2 Study Characteristics

Table 2 shows details of exercise per study. Exercise therapy interventions were grouped into four categories, including aerobic+resistance training (10 of 21 studies, 47.6%),^10^, ^11^, ^12^, ^13^, ^14^, ^15^, ^16^, ^19^, ^20^, ^34^ aerobic exercise only (3 of 21 studies, 14.3%),^21^, ^22^, ^35^ resistance training only (1 of 21 studies, 4.8%),^23^ yoga training (1 of 21 studies, 4.8%),^24^ and mixed/other exercise (5 of 21 studies, 23.8%).^5^, ^17^, ^25^, ^26^, ^27^ One study did not report the type of exercise.^18^ Within the mixed/other exercise studies, one study consisted of total body exercise and daily pelvic floor muscle exercises.

**TABLE 2:** Study characteristics

Author, year	Cancer subtype	Exercise intervention	Prehab (*n*)	Control (*n*)	Prehab mean age	Control mean age	Time at which outcomes were assessed
Barassi, 2018	Lung	3	16	16	71.3	71.8	1 week postop
Benzo, 2011	Lung	1, 2, 4	9	8	72.0	70.8	4 weeks postop
Bobbio, 2007	Lung	1, 2	11	NA	71.0	NA	
Bousquet‐Dion, 2018	Colorectal	1, 2	37	26	74.0	71.0	Preop, 4 weeks, postop, 8 weeks postop
Carli, 2020	Colorectal	1, 2	38	30	78	82	
Chen, 2017	Colorectal	1, 2	57	59	67.9	67.3	–
Dronkers, 2014	Colorectal	1, 2, 4	21	20	71.1	68.8	Preop, postop
Dunne, 2016	Colorectal Liver Mets	1	19	16	61.0	62.0	
Gillis, 2014	Colorectal	1,2	38	39	65.7	66.0	8 weeks postop
Karenovics, 2017	Lung	1	74	77	64.0	64.0	52?
Lai, 2017	Lung	1, 4	51	50	63.8	64.6	4 weeks postop
Li, 2013	Colorectal	1, 2	42	45	67.4	66.4	Preop, 4 weeks postop, 8 weeks postop
Licker, 2016	Lung	1, 2	74	77	64.0	64.0	
Mayo, 2011	Colorectal	NA	75	NA	60.0	NA	9 weeks postop
Minnella, 2018	Esophagogastric	1, 2	26	25	67.3	68.0	Preop, 4–8 weeks postop
Santa Mina, 2018	Prostate	4	37	34	61.2	62.2	4 weeks postop
Sebio Garcia, 2017	Lung	2	10	12	70.9	69.4	Postop
Singh, 2018	Rectal	1, 2	10	NA	54.4	NA	Preop, 8 weeks postop
Singh, 2017	Prostate	1, 2	10	NA	68.0	NA	Preop, 6 weeks postop
Stefanelli, 2017	Lung	1, 4	NR	NR	65.5	64.8	Preop, 8 weeks postop
West, 2015	Rectal	1	21	13	64.0	72.0	6 weeks postop
			Total sum: 676	Total sum: 547	Mean: 66.8	Mean: 67.9	

Exercise Intervention: 1 = aerobic; 2 = resistance; 3 = yoga; 4 = mixed/other; NR = not reported.

Home‐based interventions were used in 8 out of 21 studies. Supervised interventions were applied in 15 out of 21 studies. Finally, 4 of 21 studies combined supervised and home‐based exercise. Studies reported duration for the prehabilitation intervention, which included the average length of each session (hours), average number of sessions per week (frequency), and the number of weeks of prehabilitation. Reporting using frequency, intensity, time, and type (FITT) criteria is provided in Table 3. The median session duration was 0.83 h (interquartile range, IQR: 0.5–3.5 h), performed for a median of 3.3 times per week (IQR 2–10), for a median of 4 weeks (IQR 1–16). For intensity levels, five were “high” intensity, nine were “moderate,” and one was “low” intensity prehabilitation interventions, and six were not reported.

**TABLE 3:** Prehabilitation therapy characteristics, accessibility, and feasibility

Author, year	Frequency (sessions per week)	Intensity level	Weeks of ET	Avg Session duration (hr)	Supervision	Home‐based	Patients approached	Patients agreeing to participate (%)	Patients completing study (%)	GRADE
Barassi, 2018	7	Low	1	1.0	Yes	NR	NR	32	32 (100)	High
Benzo, 2011	10	Moderate	2	0.67	Yes	Yes	NR	19	19 (100)	High
Bobbio, 2007	5	High	4	1.5	Yes	No	12	12 (100)	11 (91.7)	Very low
Bousquet‐Dion, 2018	4.5	Moderate	4	0.50	Yes	Yes	88	80 (90.9)	73 (91.3)	High
Carli, 2020	7	Moderate	4	.78	Yes	Yes	120	120 (100)	110 (91.7)	High
Chen, 2017	3	NR	4	0.67	No	Yes	NR	116	116 (100)	High
Dronkers, 2014	2	Moderate	3	1.0	Yes	NR	42	42 (100)	41 (97.6)	High
Gillis, 2014	3	Moderate	4	0.83	No	Yes	104	38 (36.5)	35 (92.1)	High
Dunne, 2016	3	Moderate	4	0.50	Yes	No	97	89 (91.7)	84 (94.4)	High
Karenovics, 2017	3	High	3	0.61	Yes	No	189	164 (86.8)	156 (95.1)	High
Lai, 2017	7	High	1	1.0	Yes	NR	132	101 (76.5)	95 (94.1)	High
Li, 2013	3	NR	4	1.0	Yes	NR	NR	NR	NR	Low
Licker, 2016	2.5	High	4	.67	Yes	NR	177	164 (92.7)	151 (92.1)	High
Mayo, 2011	NR	NR	NR	NR	NR	NR	167	133 (79.6)	95 (71.4)	Very low
Minnella, 2018	4	Moderate	6	0.50	No	Yes	113	68 (60.2)	62 (91.2)	High
Santa Mina, 2018	3.5	Moderate	NR	1.0	No	Yes	185	86 (46.5)	73 (84.9)	High
Sebio Garcia, 2017	4	Moderate	8	1.0	Yes	No	68	46 (67.6)	22 (47.8)	High
Singh, 2017	2	NR	16	NR	NR	NR	17	12 (70.6)	10 (83.3)	Very low
Singh, 2018	2	NR	6	1.5	Yes	Yes	14	10 (71.4)	10 (100)	Very low
Stefanelli, 2017	5	High	3	3.5	Yes	NR	NR	NR	NR	Moderate
West, 2015	3	NR	6	0.67	Yes	No	39	39 (100)	35 (89.7)	Moderate
	Median: 3.3		Median: 4	Median: 0.83			Total sum: 1564	Total sum: 1371 (87.7)	Total sum: 1230 (89.7)	

Abbreviations: NR, not reported.

Prehabilitation interventions for the five studies included in the meta‐analysis utilized aerobic +resistance training. Home‐based interventions were used in four out of five studies.^10^, ^12^, ^15^, ^19^ Supervised interventions were applied in three out of five studies.^10^, ^13^, ^19^ Two of these studies utilized both home‐based and supervised studies.^10^, ^19^

Table 2 demonstrates the patient demographics and study characteristics of the 21 studies (published from 2007 to 2020) included in the systematic review. In total, data was included for 676 patients (mean age: 66.8 years) in the prehabilitation exercise groups and 547 patients (mean age: 67.9 years) in the control groups completing the study. The 21 studies determined the effects of prehabilitation on patients with colorectal cancer (606 patients, 49.6%),^10^, ^11^, ^12^, ^13^, ^16^, ^18^, ^19^, ^21^, ^22^, ^26^ lung cancer (485 patients, 39.7%),^14^, ^17^, ^20^, ^23^, ^24^, ^25^, ^27^, ^35^ esophagogastric tumors (51 patients, 4.4%),^15^ and prostate cancer (81 patients, 7%).^5^, ^34^

The five studies included in the meta‐analysis were published from 2013 to 2020 and studied the effects of prehabilitation on colorectal cancer (four of five)^10^, ^12^, ^13^, ^19^ and esophagogastric cancer (one of five).^15^ In total, the studies included 346 patients, with 181 patients in the prehabilitation exercise groups and 165 patients in the control groups.

### 3.3 Synthesis of results

#### 3.3.1 Objective 1: Acceptability, feasibility, and safety of prehabilitation intervention

Among 1564 patients initially approached for participation in the studies, 1371 accepted (87.7%); of these 1371 who accepted, 1230 (89.7%) completed the prescribed prehabilitation intervention. Some of the commonly reported reasons for patient refusal to partake in studies were low interest, work or time constraints, physical or medical contraindications, and access to transportation. These results are summarized in Table 3. Across all 21 studies there were no grade 3, 4, or 5 events reported. These results can be seen in Table S3.

Specifically looking at the five studies included in the meta‐analysis, the Bousquet‐Dion, Gillis, and Carli study involved patients with colorectal cancer and reported a higher percent acceptability (90.9%, 91.7%, and 100% respectively) than the Minnella study (60.2%) which investigated esophagogastric cancer. Of the 45 patients who declined to participate in the Minnella study, reasons included living too far to participate (12, 26.7%) and being too weak to exercise (33, 73.3%). However, the overall feasibility of these three studies was 339 of 357, 95.0%. Table S2 demonstrates other study‐specific outcomes of the studies evaluating prehabilitation in the 21 studies. Thirteen of the 21 measured 6MWT distance and it was found that 10 of 13 (76.9%) of the studies found a significant improvement in the 6MWT. For those who studied CPET/VO_2_, six of seven (86%) observed a significant difference, two studies measured METS/CHAMPS and both (100%) determined a significant improvement. Four of five (80%) determined a significant improvement in physical function and strength. Two of two studies (100%) observed improved mood. One of four (25%) studies found shorter length of stay or less readmission rates.

#### 3.3.2 Objective 2: Postoperative 6‐minute walk test

**FIGURE 2:**
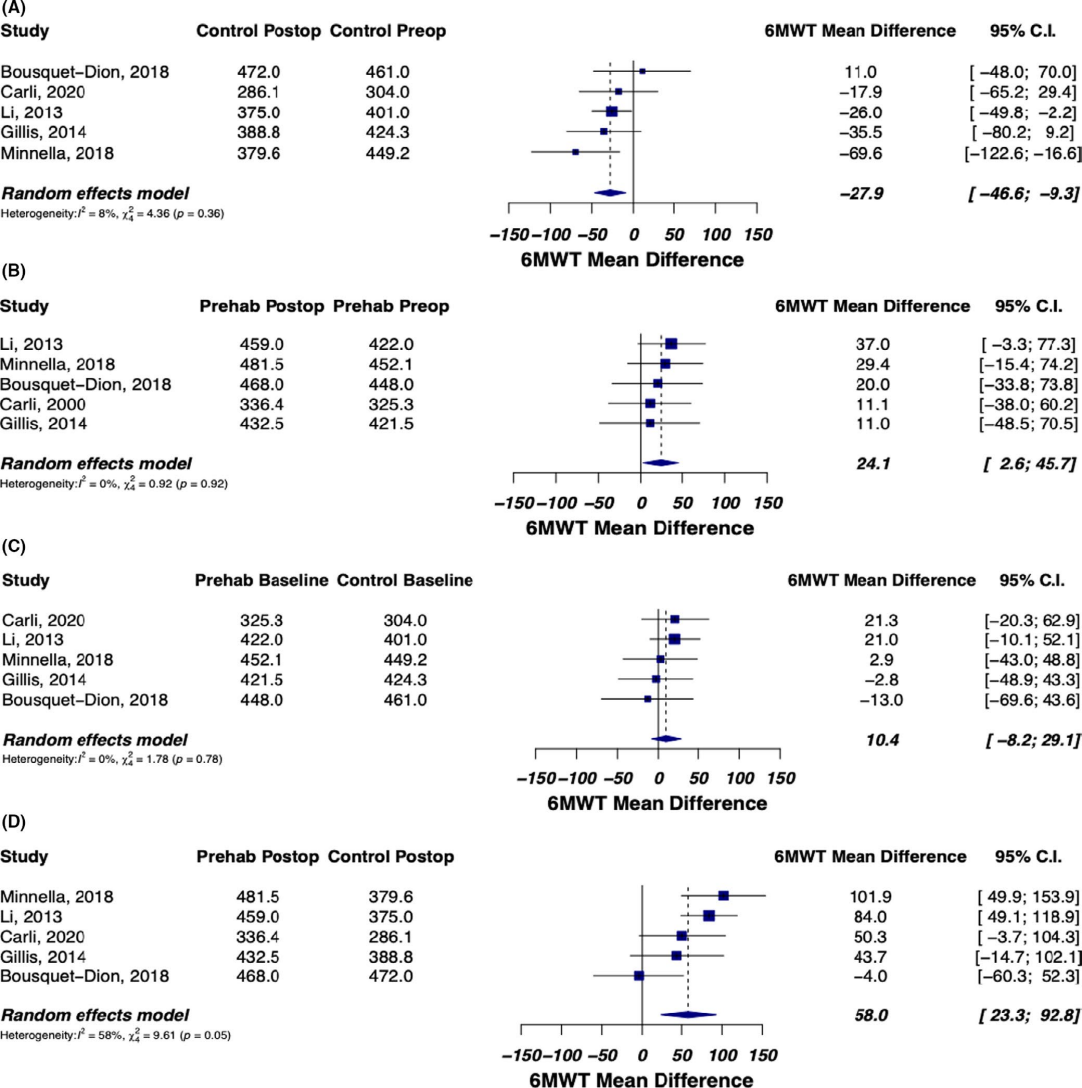
(A) Mean difference in distance (meters) walked during 6MWT for the control group at preop and postop. This was calculated by mean 6MWT distance of control group at preop – mean 6MWT of control group at postop. The results demonstrate a mean difference of +27.9 m, 95% CI: 9.3, 46.6, indicating a decrease in distance walked during 6MWT postoperatively in the control group. (B) Mean difference in distance (meters) walked during 6MWT for the prehab group at preop and postop. This was calculated by mean 6MWT distance of the prehab group at preop – mean 6MWT of the prehab group at postop. The results demonstrate a mean difference of −24.1 m, 95% CI: −45.7, −2.6, indicating an increase in distance walked during the 6MWT postoperatively in the prehab group. (C) Mean difference in distance (meters) walked during 6MWT for control and prehab at baseline (prior to surgery). This was calculated by mean 6MWT distance of control group at baseline – mean 6MWT distance of prehab group at baseline. The results indicate that the two groups (control and prehab) have the same 6MWT distance before randomization. (D) Mean difference in distance of 6MWT for control and prehab postoperatively. This was calculated by mean 6MWT distance of control group postoperatively – mean 6MWT distance of prehab group postoperatively. The results demonstrated a mean difference of −58.0 m, indicating the prehab intervention improved the 6MWT by 58.0 m. Control *n* = 135; Prehab *n* = 143

The five studies included in the meta‐analysis all objectively assessed functional outcome utilizing the 6MWT. Figure 2A,B demonstrate 6MWT distances in each of the two groups preoperatively versus postoperatively. Meta‐analysis demonstrated a mean difference (control preop – control postop) of +27.9 m, 95% CI: 9.3, 46.6, indicating that in the control group, the mean distance walked during the 6MWT decreased by an average of 27.9 m (Figure 2A). In contrast, in the prehab group, meta‐analysis demonstrated a mean difference (prehab preop – prehab postop) of −24.1 m, 95% CI: −45.7, −2.6. This indicates that in the group that received exercise prehabilitation, distance walked during the 6MWT was greater postoperatively than preoperatively. Comparing mean distance in 6MWT at baseline, there was no significant difference found in control versus prehabilitation (mean difference = −10.4; 95% CI: −29.1, 8.2, *I* ^2^ = 0%) (Figure 2C). At 4–8 weeks postoperatively, comparing the prehabilitation group to the control group, there was a significant difference in distance walked during 6MWT (mean differenc = −58.0; 95% CI: −92.8, −23.3.4, *I* ^2^ = 68%) (Figure 2D).

In the Li study, the 6MWT postoperatively demonstrated a mean distance of 375 m in the control group, while the prehabilitation group had a mean distance of 459 m (*p* ≤ 0.01). In the Minnella study, the control group had a mean 6MWT distance of 379.8 m while the prehabilitation group had a 6MWT distance of 481.5 m (*p *< 0.001). Similarly, the Gillis study, determined that functional walking capacity increased (≥20 m) in a higher proportion of the prehabilitation group compared with the control group (53% vs. 15%, adjusted *p *= 0.006) as well as a significantly higher postoperative 6MWT distance in the prehabilitation group (+23.7 m) versus the control group (−21.8 m) when compared to their respective baseline distances. However, contradictory findings were discovered in the Bousquet‐Dion study, where the prehabilitation group had a postoperative 6MWT distance of 468 m (baseline 6MWT distance: 448 m), while the control group had a 6MWT distance of 472 m (baseline 6MWT distance: 461 m). Additionally, the control group tended to be younger (*p *= 0.05) compared to the prehabilitation group. The proportion of patients aged greater than or equal to 75 years old was also lower in the control group, but not to a significant extent (23% vs. 43%, *p *= 0.098).

### 3.4 Risk of bias

Across studies, all studies had a high risk of bias for blinding of participants and personnel (performance bias). The reason for this is that it is impossible for patients to know which group they belong to given the nature of the study. All studies had a low risk of bias due to incomplete outcome data (attrition bias) or selection reporting (reporting bias). Some studies were found to have high risk of bias due to a lack of blinding of outcome assessment (detection bias). These same studies also had a high risk of bias due to poor allocation concealment (selection bias). Risk of bias can be found in Table S5.

## 4 DISCUSSION

The NCCN guidelines currently do not include any prehabilitation exercise guidelines for cancer patients. As of 2020, studies have supported prehab exercise with varying degrees. We conducted a meta‐analysis and discovered an overall patient acceptability of 87.7% and overall feasibility of 89.7%, with no significant toxicities. Meta‐analysis demonstrated a decrease in meters walked postoperatively (mean difference 27.9; 95% CI: 9.3, 46.6) in the control group and an increase in the distance walked in the prehab group (mean difference −24.1; 95% CI: −45.7, −2.6). Additionally, prehabilitation exercise can improve 6MWT distance by 58.0 m (95% CI: 23.3, 92.8) postoperatively. These data support the integration of prehabilitation into the guidelines.

Because cancer and its treatments frequently lead to disability and financial burdens, these findings may have several implications. Surgery is associated with short‐term and long‐term adverse effects, including decreased cardiorespiratory fitness, high rates of postoperative complications and mortality, emotional distress, anxiety, and poor quality of life. More specifically, surgical complications after esophagogastric surgery can impair nutritional, physical, and performance status, disabling most patients from receiving the complete sequence of neoadjuvant or adjuvant therapy and ultimately overall 5‐year survival remains poor. Therefore, optimizing perioperative functional capacity is a compelling aim in cancer patients with poor survival rates.^36^

The majority of patients studied thus far have been colorectal and lung cancer patients. Only a few studies in this analysis included cancers of prostate^5^, ^34^ or esophagogastric^15^ origins and none included breast – which accounts for about 30% of female cancers – or pancreatic cancer – which has the lowest 5‐year relative survival rate (9%).^37^ There would be great benefit investigating the effects of prehabilitation on survival in cancer patients expected to have poor survival and cancers with high prevalence. The difficulty, however, is that compliance is an arduous outcome of behavioral interventions in patients with cancer and specifically a challenge in those with poorer prognosis such as esophagogastric cancer care. This is likely given the severity of illness in his cancer patient population. Plausible explanations may be the low physical fitness of this particular population, their comorbidities, and the high rate of neoadjuvant therapy.

Francesco Carli, et al. conducted a recent randomized clinical trial that looked at the extent to which a prehabilitation program affects 30‐day postoperative complications in frail patients undergoing colorectal cancer resection compared with postoperative rehabilitation and found that the prehabilitation program did not affect postoperative outcomes. It reports that a 4‐ to 5‐week prehabilitation program may not be sufficient to increase physiological reserve preoperatively and reduce postoperative complications. This study specifically focused on the clinical outcomes of frail patients (Fried Frailty Index ≥2), outcome was measured using the Comprehensive Complication Index (CCI). The discrepancy in patients completing the prehabilitation program to the number of patients who underwent 6MWT postoperatively can be attributed to patients being lost to follow‐up. Regardless this highlights the importance of more data needed on prehabilitation to accurately ascertain the appropriate recommendations going forward.^19^

Prehabilitation aims to improve preoperative functional and physiological capacity sufficiently to enable patients to withstand surgical stress and facilitate postoperative recovery and survival. Future studies are required to investigate cancer types with overall poor prognoses in order to determine the potential benefits of prehabilitation.^38^ To our knowledge, a study comparing specific exercise types such as yoga versus aerobic and resistance has not yet been done. Studies looking at optimal length of prehabilitation to increase physiological capacity sufficiently have also not been done yet. Other prehabilitation interventions such as nutritional counselling, and anxiety reduction strategies could also be studied to determine the optimal modality of the intervention and its effect on overall oncologic outcomes. The use of 6MWT may be integrated into other models (e.g., the METSSS model) to predict survival for cancer patients.^39^

### 4.1 Study limitations

This study has several limitations. First, not all studies measured or reported necessary 6MWT data both at baseline and postoperatively, so they could not be included in the meta‐analysis. Next, it was not possible for patients to be blinded. With exercise therapy, patients know if they are getting the prehabilitation treatment and often times the clinician is aware, leading to a high risk of bias for allocation concealment and blinding of participants and personnel. Another limitation in methodology is that not all studies included were randomized. Our analysis principally included patients undergoing surgery but was not focused on patients receiving chemotherapy or radiation therapy. Additionally, evaluation of the benefit of prehabilitation in patients with other forms of cancer is still needed (breast and pancreatic). Another limitation is the lack of patient level data and language limits were imposed, as our search was limited to English. Data included is of averages and although significance is evident, no information or long‐term follow‐up exists on the level of the patient.

Additionally, some key reasons why there is heterogeneity in the meta‐analyses include: different types of cancers assessed across the trials, different types of exercise interventions assessed across trials, and prehab protocols not standardized across trials (e.g., intensity, frequency, average session times, and supervision). Prehabilitation may also have a benefit if used with certain systemic therapies (e.g., immunotherapy), as they are both postulated to help ramp up the immune response against the cancer with limited toxicity when combined with radiotherapy.^40^, ^41^

In regards to the 6MWT, it has been demonstrated as valid with test‐retest reliability in cancer patients. However, some authors suggest potential familiarity with the walking course or better pacing, leading to an artificially greater distance walked. One study demonstrated a significant 3.1% (17 m) mean increase in 6MWT distance from test to retest. Due to this, some authors suggest familiarization trials (i.e., performing a second test to establish a baseline value).^8^ This validity study has also been noted that the 6MWT may not be sensitive enough to detect effects secondary to intervention in patients with early disease who have reasonable functional capacity. Similarly, no studies have yet demonstrated correlation between 6MWT distance and overall long‐term survival. Lastly, given the risk of bias and GRADE assessment, which may have impacted the findings of the meta‐analysis, more repeated measures in larger populations, and in various cancer groups, may help to evaluate the value of a test familiarization and the clinical significance of the 6MWT.

## 5 CONCLUSION

Prehabilitation exercise for cancer patients undergoing surgery was found to be 87.7% acceptable, 89.7% feasible, and 0% significant toxicities. The meta‐analysis demonstrated statistically significant improvement in 6MWT postoperatively, with improved 6MWT distance in the prehab group by 58.0 m (95% CI: 23.3, 92.8), indicating that prehabilitation can improve postoperative functional capacity and patient survival. These results support the incorporation for prehab for cancer patients in the NCCN guidelines.

## CONFLICTS OF INTEREST

None.

## APPROVAL/DISCLOSURES

All authors have read and approved the manuscript. We have no financial disclosures. We are not using any copyrighted information, patient photographs, identifiers, or other protected health information in this paper. No text, text boxes, figures, or tables in this article have been previously published or owned by another party. This article is exempt from IRB approval.

## Supporting information

Fig S1

Supplementary Material

Table S1‐S5

## Data Availability

Data were extracted from studies—data are freely available.
