# Supplementary figures and images for: Prehabilitation exercise therapy for cancer: A systematic review and meta‐analysis
Source: Cancer Med. 2021 Jun 10;10(13):4195–205. doi: 10.1002/cam4.4021 (PMC8267161; doi:10.1002/cam4.4021)

## Slide 1
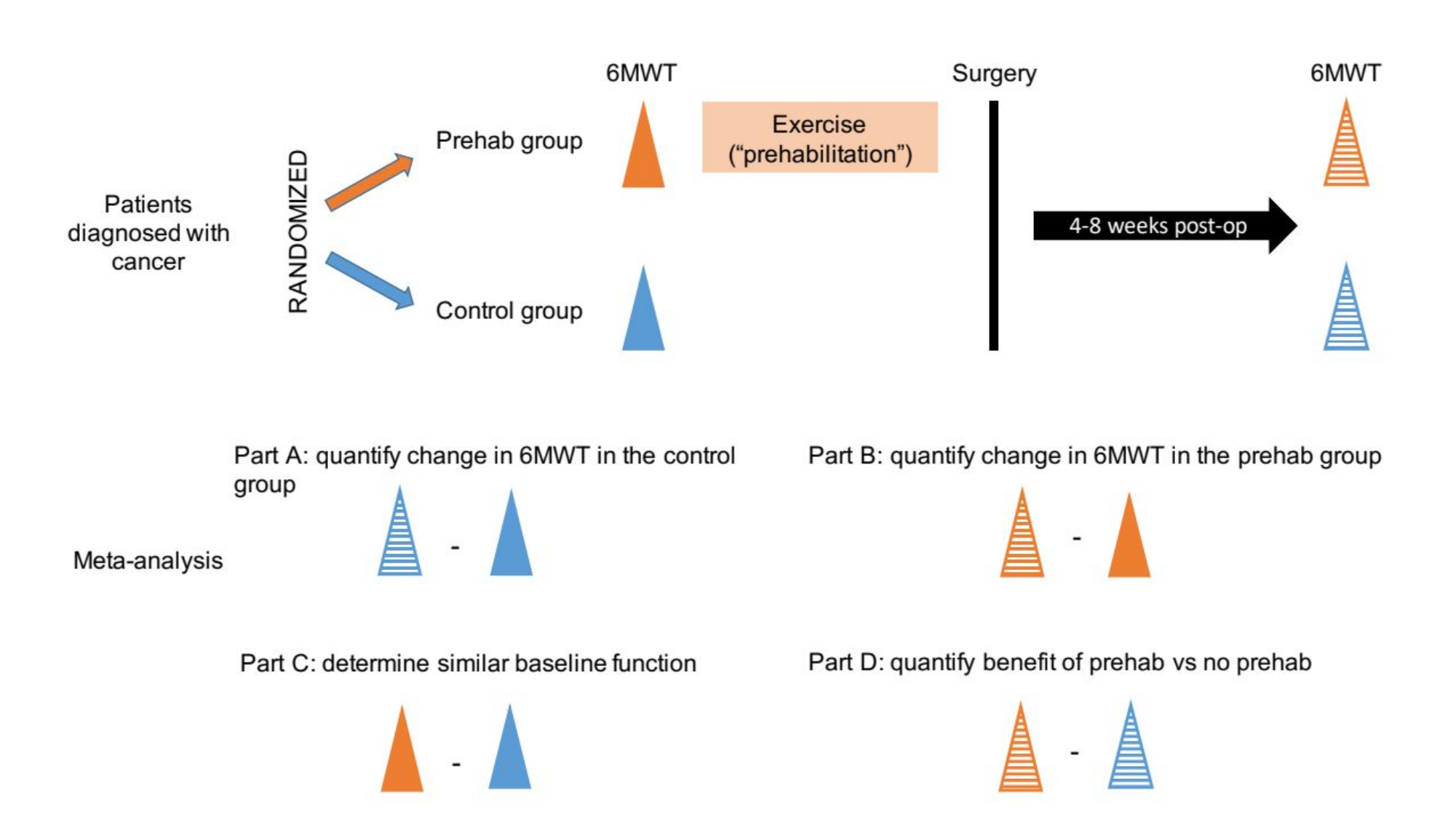

#

Supplement: Supplementary file 1 — Fig S1 [file CAM4-10-4195-s002.pptx]
