# Supplementary material for: Prehabilitation exercise therapy for cancer: A systematic review and meta‐analysis
Source: Cancer Med. 2021 Jun 10;10(13):4195–205. doi: 10.1002/cam4.4021 (PMC8267161; doi:10.1002/cam4.4021)
Supplement: Supplementary file 2 — Supplementary Material [file CAM4-10-4195-s003.docx]

**Database Search Strategy**

**Search Strategy for PubMed**

- **The original search phrase used on PubMed was**: (“cancer” or “neoplasm” or “malignancy”) and ("radiation therapy" or “radiotherapy” or “surgery”) and (“exercise” or “yoga” or "resistance training" or "weight bearing exercise” or “weight-bearing exercise” or “weightbearing exercise” or "cardiovascular training" or “cardiovascular” or "total body training" or “strength training” or “weight-lifting” or “weightlifting” or “weight training” or “pilates” or “cycling” or “aerobic” or “motion” or “movement” or “flexibility” or “walking” or “training” or “sports”) and ("prehabiliation" OR "prehab" OR “presurgical” OR “preoperative”)
- This returned the same results as “cancer" AND ("radiation therapy" OR "radiotherapy" or “surgery”) AND ("exercise" OR "yoga" OR "resistance training") AND ("prehabiliation" OR "prehab" OR “presurgical” OR “preoperative”)
- Checked two advanced options:
  - “species” chosen as “human”
  - “language” chosen as “English”
- Set the date range from 1960-2019
- Selected “Full-text” (i.e. exclude abstract)
