# Supplementary material for: Prehabilitation exercise therapy for cancer: A systematic review and meta‐analysis
Source: Cancer Med. 2021 Jun 10;10(13):4195–205. doi: 10.1002/cam4.4021 (PMC8267161; doi:10.1002/cam4.4021)
Supplement: Supplementary file 3 — Table S1‐S5 [file CAM4-10-4195-s001.docx]

# SUPPLEMENTARY MATERIAL

**Supplementary Table 1. Checklist for Meta-analyses of Observational Studies (MOOSE)**

| **Item No** | **Recommendation** | **Reported on Page No / section** |
| --- | --- | --- |
| Reporting of background should include | | |
| 1 | Problem definition | Introduction |
| 2 | Hypothesis statement | Introduction |
| 3 | Description of study outcome(s) | Methods “Outcome Measures” Paragraphs 1, 2 |
| 4 | Type of exposure or intervention used | Methods “Outcome Measures” Paragraph 2 |
| 5 | Type of study designs used | Methods “Literature Selection” Paragraph 1 |
| 6 | Study population | Introduction |
| Reporting of search strategy should include | | |
| 7 | Qualifications of searchers (eg, librarians and investigators) | Methods “Literature Selection” Paragraph 2 |
| 8 | Search strategy, including time period included in the synthesis and key words | Methods “Literature Selection” Paragraph 1 |
| 9 | Effort to include all available studies, including contact with authors | Methods “Literature Selection” Paragraph 3 |
| 10 | Databases and registries searched | Methods “Literature Selection” Paragraph 1 |
| 11 | Search software used, name and version, including special features used (eg, explosion) | Methods “Statistical Analysis” |
| 12 | Use of hand searching (eg, reference lists of obtained articles) | Methods “Literature Selection” Paragraph 2 |
| 13 | List of citations located and those excluded, including justification | Methods “Literature Selection” Paragraph 1; Figure 1 |
| 14 | Method of addressing articles published in languages other than English | Methods “Literature Selection” Paragraph 3 |
| 15 | Method of handling abstracts and unpublished studies | Methods “Literature Selection” Paragraph 3 |
| 16 | Description of any contact with authors | Methods “Literature Selection” Paragraph 3 |
| Reporting of methods should include | | |
| 17 | Description of relevance or appropriateness of studies assembled for assessing the hypothesis to be tested | Methods “Literature Selection” Paragraph 2 |
| 18 | Rationale for the selection and coding of data (eg, sound clinical principles or convenience) | Methods “Data Abstraction and Analysis” |
| 19 | Documentation of how data were classified and coded (eg, multiple raters, blinding and interrater reliability) | Methods “Literature Selection” Paragraph 3 |
| 20 | Assessment of confounding (eg, comparability of cases and controls in studies where appropriate) | Discussion “Study Limitations” |
| 21 | Assessment of study quality, including blinding of quality assessors, stratification or regression on possible predictors of study results | Methods “Literature Selection” Paragraph 3 |
| 22 | Assessment of heterogeneity | Methods “Statistical Analysis” |
| 23 | Description of statistical methods (eg, complete description of fixed or random effects models, justification of whether the chosen models account for predictors of study results, dose-response models, or cumulative meta-analysis) in sufficient detail to be replicated | Methods “Statistical Analysis” |
| 24 | Provision of appropriate tables and graphics | Results |
| Reporting of results should include | | |
| 25 | Graphic summarizing individual study estimates and overall estimate | Figure 2. |
| 26 | Table giving descriptive information for each study included | Table 2, 3 |
| 27 | Results of sensitivity testing (eg, subgroup analysis) | N/A |
| 28 | Indication of statistical uncertainty of findings | Discussion “Study Limitations” |

| **Supplementary Table 2: Other Outcomes of Studies Evaluating Prehabilitation** | | | | | | |  |  |  |  |  |
| --- | --- | --- | --- | --- | --- | --- | --- | --- | --- | --- | --- |
|  |  |  | **Statistically significant improvement in patient** | | | | | | | | |
| **Author, Year** | **Cancer subtype** | **Exercise intervention** | **6MWT** | **Post-op pulmonary complications** | **CPET/ VO2** | **METS/ CHAMPS** | | **Physical function/ strength** | **Mood/ depression/ anxiety** | **QOL** | **Length of stay/ readmission rate** |
| **Barassi, 2018** | Lung | Yoga | NR | NR | NR | NR | | Yes | NR | NR | NR |
| **Benzo, 2011** | Lung | Aerobic/Resistance/Other | NR | Yes | NR | NR | | NR | NR | NR | No |
| **Bobbio, 2007** | Lung | Aerobic/Resistance | NR | NR | Yes | NR | | NR | NR | NR | NR |
| **Bousquet-Dion, 2018** | Colorectal | Aerobic/Resistance | No | NR | NR | Yes | | NR | NR | NR | NR |
| **Carli, 2020** | Colorectal | Aerobic/Resistance | No | NR | NR | NR | | NR | No | NR | No |
| **Chen, 2017** | Colorectal | Aerobic/Resistance | Yes | NR | NR | Yes | | NR | NR | NR | NR |
| **Dronkers, 2014** | Abdominal | Aerobic/Resistance/Other | NR | No | NR | NR | | Yes | NR | NR | NR |
| **Dunne, 2016** | Colorectal Liver Mets | Aerobic | NR | No | Yes | NR | | NR | NR | Yes | NR |
| **Gillis, 2014** | Colorectal | Aerobic/Resistance | Yes | NR | NR | NR | | NR | NR | NR | No |
| **Karenovics, 2017** | Lung | Aerobic | NR | NR | No | NR | | NR | NR | NR | NR |
| **Lai, 2017** | Lung | Aerobic/Other | Yes | Yes | Yes | NR | | NR | NR | NR | NR |
| **Li, 2013** | Colorectal | Aerobic/Resistance | Yes | NR | NR | NR | | NR | Yes | NR | NR |
| **Licker, 2016** | Lung | Aerobic/Resistance | Yes | Yes | Yes | NR | | NR | NR | NR | Yes |
| **Mayo, 2011** | Colorectal | NA | Yes | NR | NR | NR | | NR | NR | NR | NR |
| **Minnella, 2018** | Esophogastric | Aerobic/Resistance | Yes | NR | NR | NR | | NR | NR | NR | No |
| **Santa Mina, 2018** | Prostate | Mixed/Other | Yes | NR | NR | NR | | NR | Yes | NR | NR |
| **Sebio Garcia, 2017** | Lung | Resistance | No | NR | NR | NR | | Yes | NR | NR | NR |
| **Singh, 2018** | Rectal | Aerobic/Resistance | Yes | NR | NR | NR | | No | NR | NR | NR |
| **Singh, 2017** | Prostate | Aerobic/Resistance | Yes | NR | NR | NR | | Yes | NR | NR | NR |
| **Stefanelli, 2017** | Lung | Aerobic/Other | NR | NR | Yes | NR | | NR | NR | NR | NR |
| **West, 2015** | Rectal | Aerobic | NR | NR | Yes | NR | | NR | NR | NR | NR |
| **NR = not reported** | | | | | | | | | | | |

| **Supplementary Table 3: Safety and Adverse Events** | | | | | |
| --- | --- | --- | --- | --- | --- |
| **Author, Year** | **Cancer subtype** | **Total # Adverse Events**  **(Grades 3-5)** | **Grade 3** | **Grade 4** | **Grade 5** |
| **Barassi, 2018** | Lung | 0 | 0 | 0 | 0 |
| **Benzo, 2011** | Lung | 0 | 0 | 0 | 0 |
| **Bobbio, 2007** | Lung | 0 | 0 | 0 | 0 |
| **Bousquet-Dion, 2018** | Colorectal | NR | NR | NR | NR |
| **Carli, 2020** | Colorectal | 0 | 0 | 0 | 0 |
| **Chen, 2017** | Colorectal | 0 | 0 | 0 | 0 |
| **Dronkers, 2014** | Abdominal | 0 | 0 | 0 | 0 |
| **Dunne, 2016** | Colorectal Liver Mets | 0 | 0 | 0 | 0 |
| **Gillis, 2014** | Colorectal | NR | NR | NR | NR |
| **Karenovics, 2017** | Lung | 0 | 0 | 0 | 0 |
| **Lai, 2017** | Lung | NR | NR | NR | NR |
| **Li, 2013** | Colorectal | NR | NR | NR | NR |
| **Licker, 2016** | Lung | 0 | 0 | 0 | 0 |
| **Mayo, 2011** | Colorectal | NR | NR | NR | NR |
| **Minnella, 2018** | Esophogastric | 0 | 0 | 0 | 0 |
| **Santa Mina, 2018** | Prostate | 0 | 0 | 0 | 0 |
| **Sebio Garcia, 2017** | Lung | 0 | 0 | 0 | 0 |
| **Singh, 2018** | Rectal | 0 | 0 | 0 | 0 |
| **Singh, 2017** | Prostate | 0 | 0 | 0 | 0 |
| **Stefanelli, 2017** | Lung | NR | NR | NR | NR |
| **West, 2015** | Rectal | 0 | 0 | 0 | 0 |
| **NR = not reported** |  |  |  |  |  |

**Supplementary Table 4: Location of Studies**

| **Author, Year** | **Location of the Study** |
| --- | --- |
| **Barassi, 2018** | Carlo Bo University (Urbino, Italy) |
| **Benzo, 2011** | Mayo Clinic |
| **Bobbio, 2007** | University Hospital of Parma (Parma, Italy) |
| **Bousquet-Dion, 2018** | McGill University Health Center (Montreal, Canada) |
| **Carli, 2020** | McGill University Health Center (Montreal, Canada) |
| **Chen, 2017** | McGill University Health Center (Montreal, Canada) |
| **Dronkers, 2014** | Gelderse Vallei Hospital (Ede, Netherlands) |
| **Dunne, 2016** | Aintree Unviersity Hospital (Liverpool, UK) |
| **Gillis, 2014** | McGill University Health Center (Montreal, Canada) |
| **Karenovics, 2017** | University of Geneva and the Hospital of Valaiis |
| **Lai, 2017** | West China Hospital Sichuan University (Chengdu, China) |
| **Li, 2013** | McGill University Health Center (Montreal, Canada) |
| **Licker, 2016** | University Hospital of Geneva (Geneva, Switzerland) |
| **Mayo, 2011** | McGill University Health Center (Montreal, Canada) |
| **Minnella, 2018** | McGill University Health Center (Montreal, Canada) |
| **Santa Mina, 2018** | McGill University Health Center (Montreal, Canada) and University Health Network (Toronto, Canada) |
| **Sebio Garcia, 2017** | University of A Coruña, A Coruña, Spain |
| **Singh, 2017** | University of Western Australia, |
| **Singh, 2017** | University of Western Australia, |
| **Stefanelli, 2017** | AORN Dei Colli ‘Monaldi Hospital’, (Naples, Italy) |
| **West, 2015** | University Hospital Southampton NHS Foundation Trust |

| **Supplementary Table 5: Risk of Bias** | | | | | | |
| --- | --- | --- | --- | --- | --- | --- |
| **Author, Year** | **Random sequence generation (selection bias)** | **Allocation concealment (selection bias)** | **Blinding of participants and personnel (performance bias)*** | **Blinding of outcome assessment (detection bias)** | **Incomplete outcome data (attrition bias)** | **Selection reporting (reporting bias)** |
| **Barassi, 2018** | Low risk | Low risk | High risk | Low risk | Low risk | Low risk |
| **Benzo, 2011** | Low risk | Low risk | High risk | Low risk | Low risk | Low risk |
| **Bobbio, 2007** | High risk | High risk | High risk | High risk | Low risk | Low risk |
| **Bousquet-Dion, 2018** | Low risk | Low risk | High risk | Low risk | Low risk | Low risk |
| **Carli, 2020** | Low risk | Low risk | High risk | Low risk | Low risk | Low risk |
| **Chen, 2017** | Low risk | Low risk | High risk | Low risk | Low risk | Low risk |
| **Dronkers, 2014** | Low risk | Low risk | High risk | Low risk | Low risk | Low risk |
| **Dunne, 2016** | Low risk | Low risk | High risk | Low risk | Low risk | Low risk |
| **Gillis, 2014** | Low risk | Low risk | High risk | Low risk | Low risk | Low risk |
| **Karenovics, 2017** | Low risk | Low risk | High risk | Low risk | Low risk | Low risk |
| **Lai, 2017** | Low risk | Low risk | High risk | Low risk | Low risk | Low risk |
| **Li, 2013** | Low risk | High risk | High risk | High risk | Low risk | Low risk |
| **Licker, 2016** | Low risk | Low risk | High risk | Low risk | Low risk | Low risk |
| **Mayo, 2011** | Low risk | High risk | High risk | Unclear | High risk | Low risk |
| **Minnella, 2018** | Low risk | Low risk | High risk | Low risk | Low risk | Low risk |
| **Santa Mina, 2018** | Low risk | Low risk | High risk | Low risk | Low risk | Low risk |
| **Sebio Garcia, 2017** | Low risk | Low risk | High risk | Low risk | Low risk | Low risk |
| **Singh, 2018** | High risk | High risk | High risk | Unclear | Low risk | Low risk |
| **Singh, 2017** | High risk | High risk | High risk | Unclear | Low risk | Low risk |
| **Stefanelli, 2017** | Low risk | Low risk | High risk | Unclear | Low risk | Low risk |
| **West, 2015** | Low risk | High risk | High risk | Low risk | Low risk | Low risk |
| * For all of these studies, patients and clinicians knew which group participants were randomized do. Group who received exercise treatment received more care than the group who did not undergo prehabilitation exercise. | | | | | | |
